# Medical Expert Testimony

**Published:** 1897-01

**Authors:** B. M. Worsham

**Affiliations:** Superintendent Texas Insane Asylum, Austin, Texas


### TEXAS MEDICAL JOURNAL.

ESTABLISHED JULY, 1885.

PUBLISHED MONTHLY.—SUBSCRIPTION $2.00 A YEAR.

VOL. XII. AUSTIN, JANUARY, 1896. NO. 7.

### Original Contributions.

For the Texas Medical Journal.

### MEDICAL EXPERT TESTIMONY.

BY B. M. WORSHAM, M. D.,
Superintendent Texas Insane Asylum, Austin, Texas.

[Read before the Austin District Medical Society December 24, 1896, also before the Ellis County Medical Society.]

GENTLEMEN OF THE ASSOCIATION:—The law in this, and many other States of this country, forbidding that any person of unsound mind be placed upon trial for crime before the courts, and the frequent attempts on the part of the attorneys to make this plea for their clients, renders it very important that physicians be prepared to give testimony intelligently and without so much variation in cases of this character. The legal profession has taken advantage of this splendid loophole of escape for so many criminals, and has studied the subject well. We find in every city or town, lawyers who are well posted on medico-legal questions, and are fully prepared to cause the doctor who may chance to oppose them, much trouble and humiliation upon the witness stand. It can not be denied that the way in which so many of our professiou acquit themselves on the witness stand, is a most potent factor in inviting criticism and bringing ridicule upon medicine as a science, and we, as medical men, are forced to confess that such criticism is, in a measure, merited.

The object of this paper, therefore, is to try to excite more interest among general practitioners on this particular subject.

When a physician is called upon to testify as to an individual's sanity, or insanity, and is placed upon the witness stand without having previously studied the case, he finds himself in a peculiarly embarrassing position. It is expected of him to form an opinion in the case from his observation of the person while on trial, and from the meager history given by witnesses who testify in the case. Under such circumstances it is utterly impossible for any physician to make a diagnosis free from doubt. Nearly, if not quite all, of the witnesses in such cases have some personal feeling, either for, or against the person being tried, and their statements are always colored to suit their feelings and individual opinions in the case, hence, a clinical history obtained from such a source is not at all reliable, and no physician (in the opinion of the writer) should attempt to give a positive opinion based upon such a history. Of course it must be understood that in cases where the insanity is so palpable that the ordinary layman could readily detect it, physicians are seldom, if ever, called upon to testify. It is the doubtful and obscure cases that furnish, "the battle ground upon which forensic battles are fought."

In order to give an opinion with any degree of accuracy, as complete a history of the case as possible should be obtained, from the most reliable source, before the study of the case begins. It is impossible to fix any standard of sanity, by which we can measure humanity, for no two person possess the same intellectual capabilities, and hence the importance of studying each case from the standpoint of the normal condition of the individual.

The legal definition of insanity, in our State, in my opinion, is not broad enough to protect the irresponsible, but we are not responsible for the law, and when we are called upon to testify as medical experts, we should be able to testify in accord with the most advanced and established facts of our prosession. Savage says "From a physicians' standpoint of view, insanity has to be considered as a disease of the brain, or a disease of the mind quite apart from any consideration of responsibility whatever." The mere knowledge of right and wrong, may and does exist in many persons, who are driven by the suggestions of a diseased imagination to commit crime. If we find that the accused possesses a faulty heredity, that point should be well considered, for it is now conceded by all alienists that when an individual is tainted with the tendency to insanity, such tendency is a perpetual menance to the intellectual stability of the individual.

A great deal of stress has been placed upon the physiognomy and the various stigmata of the insane, and it is a fact, that anatomical anomalies do exist in a very large per cent. of cases of insanity, but it is not necessary to make mention here of all the different marks of degeneracy noted by the various observers, who have given this particular point special study, for many of them are immaterial, inasmuch as they are frequently found with persons whose mental equilibrium has never been the least disturbed. Whenever marked cranial asymmetry is found in a person, sane or insane, it is the result of faulty development; is usually coupled with neurotic family history, and marks the person as a degenerate, and hence an easy victim of insanity. It must be understood, however, that the insane or neurotic family history and unmistakable evidences of degeneracy may exist in a person, and still that person be sane in the ordinary acceptation of the term; hence it has been very properly said that every man is to be considered sane or insane, in relation to himself alone.

Take the person who, by faulty ancestral predisposition, or by any arrest of development, has been endowed with an inferior intellect, if his intellectual capabilities are of high enough order or type to entitle the person to be classed above the imbecile or congenital insane, with a sufficient degree of reason to meet the ordinary demands of our social laws, then he is considered a person possessing an individuality capable of sustaining himself in the struggle for existence, and is therefore responsible for his acts. Maudsley says: "Some persons exhibit eccentricities of thought, feeling and conduct, which, not reaching the degree of positive insanity, nevertheless make them objects of remark in the world, and cause difficulty sometimes when the question of legal or moral responsibility is concerned. They are so unlike other people in their feeling and thoughts, and do such odd things, that they are thought to have a strain of madness in them; they have what may be called the insane temperament."

It is a very important point to remember that after the insane or degenerate temperament has been established to our satisfaction, there still exists a wall, so to speak, however thin and delicate it may be, separating this condition from true insanity. The physician should be ready to admit that a person possessing such a temperament is not a proper person, i. e., such a basis does not guarantee perfect stability of thought and character, still he cannot say that such a person is insane, for when iusanity does occur in such cases there is a new element added to the primitive or faulty basis.

While in all cases of insanity, no regular chain of symptoms is to be found in common with the different classifications, still we should be able to detect, or note the absence of, some of the most noted features of mental unsoundness. I shall not attempt to enumerate here the symptoms of the different forms of insanity; the are by far too numerous, and they are so variable. In the very incipiency of all forms of insanity, there is a change in the character of the individual, and in many instances, this change of character may exist long before the mental integrity can be questioned. In some cases it requires a carefully outlined history to detect such a change of character. In a man whose sanity has never been questioned, a change of character should be conclusively proven before we should venture an opinion that he is insane.

In cases of insanity where moral deficiency seems to be the principal or most prominent symptom, Maudsley's opinion is endorsed by nearly all the highest authorities when he says: "In the previous history of the patient, there will be evidence of a sufficient cause of disease having been followed by an entire change of manner, feeling and acting."

The term moral insanity, as first described by Prichard, and subsequently much discussed in medico-legal cases, is undoubtedly misleading, and has no place among the different classifications of insanity. It is a fact, however, that in many cases of insanity, moral deficiencies in different ways constitute the most prominent symptoms, but aside from cases where imbecility is certainly present, acts of moral turpitude should be accompanied by other symptoms and physical conditions before insanity can be established. Therefore, when a change of character has been established, and we can note the absence of motive in any part of the conduct, then we have the authority on our side when we say that, there has been a departure from the normal condition, and the person is therefore insane.

A very large per cent. of the criminals belong to the class of "insane temperament," and such great men as Lombroso and others, have tried to prove their irresponsibility, and have given much study in trying to produce facts and argument that will educate the people up to the point of trying to correct, instead of punishing in such cases.

The recent celebrated Burt trial in this city, furnishes an important case in point. The fact that he murdered his wife and two little children in the most brutal way, and without a known motive, would naturally suggest the possibility of insanity. The facts as shown by the evidence in the case, were that his grandfather was insane for a number of years, and at the time of his death was the victim of terminal dementia, that his mother had an attack of insanity, caused by the puerperal state, at the times of his birth, that he had one or more aunts who were the subjects of frequent and severe attacks of neuralgia and hysteria, and that several cousins were epileptics. All this would prove that he had undoubtedly inherited the insane tendency, but the only evidence worthy of note, that would tend to prove the vice of his organization, was the fact that he was a thief from early childhood. He had no noticable physical evidences of degeneracy; never in his history had he manifested any "psychical stigma," not even being considered eccentric. No evidences of excesses in any way, his habits always above the average, and his honesty was all that had ever been questioned up to the time he committed this terrible crime.

The writer saw him, and had an hour or more conversation with him shortly after his capture, and some two or three months before his trial. At that time he did not manifest the slightest evidence of depression or remorse. He refused to discuss his case with me, but talked pleasantly upon other subjects. When I left him, noting the absence of depresssion or remorse under such terrible circumstances, and without the knowledge of the slightest motive for the commission of the crime, I was in some doubt about his sanity. I was asked to examine him again, just a day or so before the trial; and in company with two other medical men, I called to see him at the jail. At this visit he was told who we were and the object of our visit, but duriug my first visit he never found out whom I was. His conduct during my second visit was so changed from what it was at my first visit, and so different from any I had ever seen in any form of insanity, my convictions were, at that time, that he was simulating. The next time I saw him was when he was brought before the court for trial. He then assumed a condition of profound stupor or coma, and seemed to take no notice of what was going on. He maintained this position with some regularity, until the close of the trial.

Taking the history of the case, obtained from the witnesses on the stand, and from all the outside facts that I could gather, I failed to note where there had ever been a change in his character, from the beginning, up to the commission of the crime; and being thoroughly convinced that he was acting, before the court and jury, what he conceived to be the acts of a madman, together with other points of minor interests in the case, I gave it as my opinion, that if sanity had ever existed with him, he had not departed from his normal standard, and therefore was not insane. I am willing to admit that the alleged motive, viz.: "That he might sell the household goods in order to raise money, which he badly needed to defray his expenses to some place of hiding from the cases which were soon to come up for trial against him for forgery, and that his wife was not willing for him to sell all that they possessed and leave her destitute," etc., is out of all proportion to such a crime, and his true motive may never be known; but my opinion was based upon the facts as I saw them, and I believe that the opinion to the effect that he was simulating at the time of the trial, has been fully proved to be correct. After his trial was completed, and his sentence was announced, he admitted to the sham he tried to play, and is now in his usual state of mind. It may be true, however, and perhaps we may never know positively, but that persons possessing such vices of organization, as the proof shows in this case, are never responsible beings, from the fact that they "cannot contemplate things in the white light of calm understanding, but see them in colors of the distempered feelings."

